# Effects of climatic and environmental factors on mosquito population inferred from West Nile virus surveillance in Greece

**DOI:** 10.1038/s41598-023-45666-3

**Published:** 2023-11-01

**Authors:** Federico Ferraccioli, Nicola Riccetti, Augusto Fasano, Spiros Mourelatos, Ioannis Kioutsioukis, Nikolaos I. Stilianakis

**Affiliations:** 1 European Commission, Joint Research Centre (JRC), Via E. Fermi 2749, 21027 Ispra, VA Italy; 2 EcoDevelopment SA, 57010 Filyro, Thessaloníki, Greece; 3 Department of Physics, University of Patras, 26504 Rio, Greece; 4 Department of Biometry and Epidemiology, University of Erlangen-Nuremberg, Waldstraße 6, 91054 Erlangen, Germany; 5 Present Address: Department of Statistical Sciences, University of Padova, Via C. Battisti 241, 35121 Padua, PD Italy; 6 Present Address: Department of Statistics, Catholic University of the Sacred Heart, Largo A. Gemelli, 20123 Milan, MI Italy

**Keywords:** Climate sciences, Ecology

## Abstract

Mosquito-borne diseases’ impact on human health is among the most prominent of all communicable diseases. With limited pool of tools to contrast these diseases, public health focus remains preventing mosquito-human contacts. Applying a hierarchical spatio-temporal Bayesian model on West Nile virus (WNV) surveillance data from Greece, we aimed to investigate the impact of climatic and environmental factors on Culex mosquitoes’ population. Our spatio-temporal analysis confirmed climatic factors as major drivers of WNV-transmitting-Culex mosquitoes population dynamics, with temperature and long periods of moderate-to-warm climate having the strongest positive effect on mosquito abundance. Conversely, rainfall, high humidity, and wind showed a negative impact. The results suggest the presence of statistically significant differences in the effect of regional and seasonal characteristics, highlighting the complex interplay between climatic, geographical and environmental factors in the dynamics of mosquito populations. This study may represent a relevant tool to inform public health policymakers in planning preventive measures.

## Introduction

Mosquitoes play a major role in transmitting pathogens to humans, being the vector of very common and geographically widespread diseases such as Malaria, Yellow Fever, Dengue, Zika, Chikungunya, and West Nile virus disease^1–3^. The latter disease is caused by West Nile virus (WNV): a single-strained positive-polarity Flavivirus, which is transmitted mostly by mosquitoes from genus *Culex*^1,4^. WNV is maintained in an enzootic circle between these mosquitoes and birds, with other vertebrates—especially human and horses—as occasional and dead-end hosts^1,4^. Although often a- or pauci-symptomatic, the disease resulting from WNV infections in humans might develop in a severe neurological condition (West Nile neuro-invasive disease or WNND), which can present either as encephalitis, meningitis, acute flaccid paralysis or a combination of these three^4–6^. This neuro-invasive disease often presents with high proportion of patients needing to be hospitalized^7–9^, as well as with long-term symptoms and sequelae^1,5,7,10–16^, and a case fatality risk that ranges between 13% and 18%, depending on the gravity of the symptoms^9^. In addition, WNV outbreaks are becoming more common and worldwide distributed each year, being to date considered as one of the leading causes of infectious disease encephalitis^4^.

To date, no specific therapeutical approach such as vaccination or treatment is available to contrast this disease^1,6^; hence, public health focus remains the prevention of mosquito-human contact. Public health policy makers as well as researchers often rely on mathematical and/or statistical models of mosquito population abundance and virus transmission to determine proper interventions and the corresponding optimal times aimed at preventing the spread of WNV. However, these models, which are often based on climatic factors, are potentially hindered by uncertainty, particularly in the estimation of the mosquito population.

This because, while there is a general agreement that these climatic factors play an important role in determining mosquito population dynamics, with temperature affecting, e.g., mosquito immature development, adult size, and survival^17–19^, many of these results were observed in laboratory conditions and may not be immediately transferred to field and real-world settings. For these reasons we considered of relevance to address these limitations by introducing a new model that explores the dynamics of mosquito populations at trap level, unlike previous studies that focused on regional or sub-regional level. This approach provides benefits such as insights into mosquito populations involved in WNV transmission at a finer scale.

## Results

**Figure 1:**
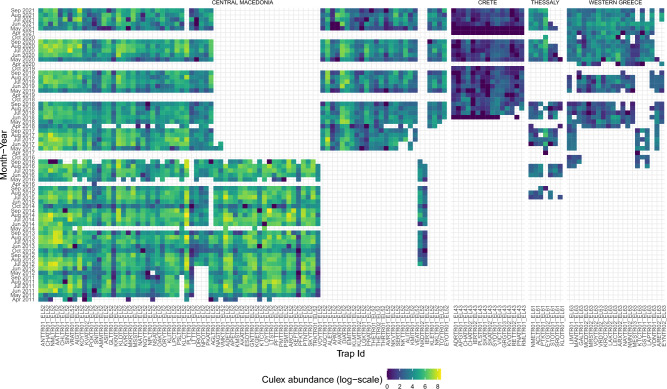
Culex mosquito abundance in logarithmic scale, stratified by trap (columns) and month-year (rows).

**Figure 2:**
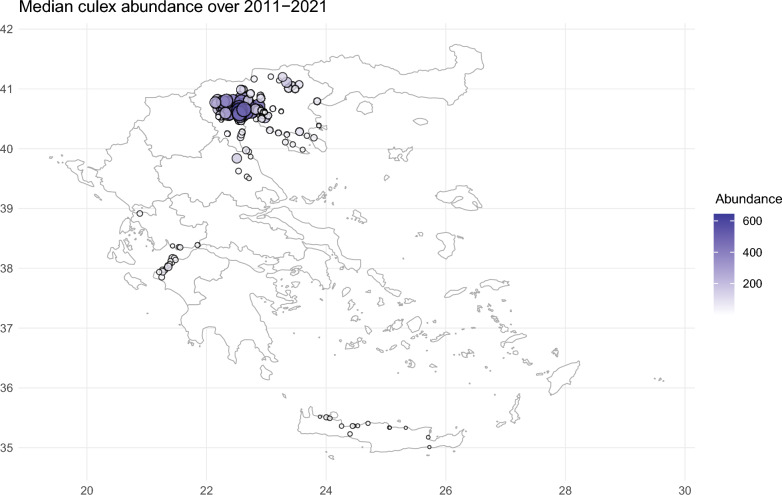
Distribution of traps over the Greece territory and median culex abundance highlighted with color (darker shades corresponds to higher abundances) and size (larger areas corresponds to higher abundances). The map has been created using the package ggplot2 within the R software (https://www.r-project.org/, version 4.3.1), with official boundaries obtained from Eurostat (https://ec.europa.eu/eurostat/web/gisco/geodata).

Average monthly mosquito abundance data from 2011 to 2021 were available from 126 unique traps (Fig. 1). These traps are part of the Greek WNV surveillance program and, therefore, are distributed on the territory favoring the regions with higher presence of WNV human cases (e.g. Central Macedonia) (see Fig. 2). More precisely, the number of available traps is 84 for Central Macedonia region, 15 for Crete, 7 for Thessaly and 20 for Western Greece (see Figure 1). Note that the locations and number of traps change over the years, this is further addressed in the "Discussion" section. The average amount of captured mosquito is 222.3 (SD: 391.32, range 0–4,758) with a standard deviation of 391.31.

Mosquitoes captures were higher in Central Macedonia, and less/non-present in Thessaly, Western Greece and Crete (in decreasing order). Similarly, mosquitoes captures were higher in the central months from June to August, and less/non-present in the months April, May and September, October (see Supplementary Table S1 for the summary statistics for all the traps).

The WNV surveillance system in Greece has been in developing since its beginning in 2011. In the first years, the number of mosquito collections were lower, with values (603, 862, 467, 557, 556, 703, 997) for the years ranging from 2011 to 2017. In the years 2018–2021 the values were significantly higher, more precisely (1652, 1364, 1665, 1640), respectively. Data availability is therefore quite unbalanced at trap site within the whole time period: traps set at specific municipalities e.g., Aitoliko, Chalastra, Nea Malgora, Agios Athanasios, Chalkidonia, Sindos, and Vrachia are the ones with most data available, whereas the ones set at e.g., Kileler, Eyaggelismos, and Kastri Loythro, are the ones with the least data available (Fig. 1).

### Effect of climatic and environmental factors

**Figure 3:**
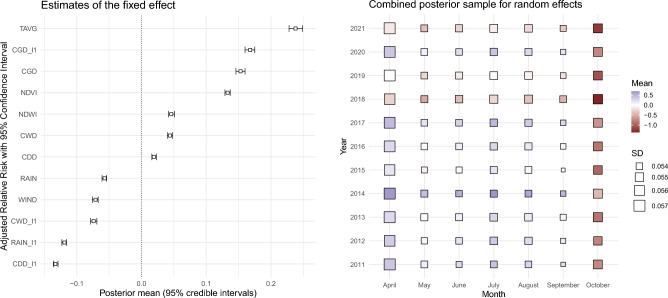
Left: Estimated posterior mean for the fixed effect and corresponding $95\%$ Bayesian credible intervals for potential predictors of mosquitoes abundance, reported from the most positive association to the most negative. Considered factors were: average temperature (TAVG), consecutive number of growth days in previous month (CGD_l1), consecutive number of growth days in current month (CGD), vegetation abundance index (NDVI), water abundance index (NDWI), consecutive number of wet days (CWD), consecutive number of dry days (CDD), average rainfall (RAIN), average wind speed (WIND), consecutive number of wet days in previous month (CWD_l1), average rainfall in previous month (RAIN_l1), and consecutive number of dry days in previous month (CDD_l1). Rigth: Combined posterior mean and standard deviation (SD) for the random effects for month and year. Different mean values are reported with different colours, whereas different SDs are reported with different sizes of the squares.

The fixed effects, i.e. the linear effect of the covariates on the mosquito abundance (in log-scale) are shown in Fig. 3. On the left side are presented the posterior estimates for the coefficients of the covariates, together with the 95% posterior credibility interval (PCI). Note that the covariates are standardized, therefore the magnitude of their effects is comparable between one another. The average monthly temperature has the strongest positive effect on mosquito abundance, with a mean estimate of 0.238 (SD: 0.0053, PCI: 0.228–0.249). Similarly, the consecutive number of grow days (CGD) in the concurrent and previous month have a positive effect, with mean estimates of 0.153 (SD: 0.0037, PCI: 0.146–0.160) and 0.168 (SD: 0.0039, PCI: 0.160–0.175), respectively. Additionally, positive effects are observed for NDWI and NDVI, with mean estimates of 0.046 (SD: 0.0023, PCI: 0.042–0.051) and 0.133 (SD: 0.0021, PCI: 0.129 – 0.137), respectively. Other variables, including consecutive number of dry days (CDD) and consecutive number of wet days (CWD), also have a positive impact on mosquito population, with mean estimates of 0.019 (SD: 0.0018, PCI: 0.016–0.023) and 0.044 (SD: 0.0018, PCI: 0.040–0.048), respectively.

Conversely, the amount of rainfall, both in the concurrent and previous months, is associated with a negative effect to *Culex* abundance, with mean estimates of − 0.057 (SD: 0.0017, PCI: − 0.061 to − 0.054) and − 0.119 (SD: 0.0017, PCI: − 0.123 to − 0.116), respectively. Consecutive wet and dry days in the previous month also have a negative effect, with mean estimates of − 0.074 (SD: 0.0023, PCI: − 0.079 to − 0.069) and − 0.133 (SD: 0.0018, PCI: − 0.136 to − 0.129), respectively. Finally, the average wind speed is also associated with a negative impact on mosquito abundance, with a mean estimate of − 0.071 (SD: 0.0022, PCI: − 0.076 to − 0.67).

### Spatial and temporal random effect

We now consider the results of the hierarchical temporal (month and calendar year) and spatial component. The posterior mean for the temporal effect for month is positive in the period from April to September, with increasing values from May to August and decreasing values from August to October. A possible explanation may include differences in larvae development and spraying procedures. As for the temporal effect for calendar year, there is no clear patterns except for a noticeable lower value for the years 2018 and 2021. This suggest an unexpected deviation in mosquito abundance that is not consistent with the effect of the covariates and the spatial effect, and it is likely due to external factors not considered in the model. The combined temporal random effect by month and year (Fig. 3, right panel) depicts a more clear picture. The overall pattern is consistent across most years, with higher positive values observed in the central months of July and August, excluding April and October. In addition, there is a noticeable increase in variability in the months of April and October, likely due to the limited number of observations in those months. The posterior means for April are mostly positive, while October shows the opposite trend with all negative values. This deviation may be attributed to the model’s smoothing effect, which prioritizes adhering to the behavior of central months over a more precise estimation in April and October (see Supplementary Figure S1 for the posterior distribution of the spatial and temporal effects).

**Figure 4:**
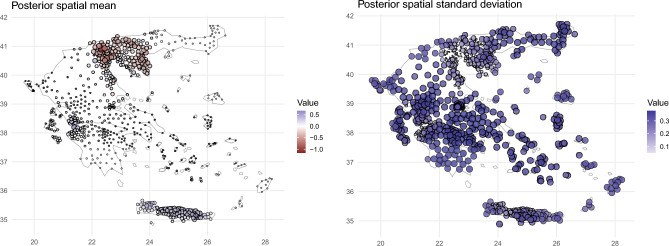
Left: Estimated posterior mean. Right: Estimated posterior standard deviation for the spatial random effect.

The posterior mean for the spatial random effect shows a very smooth behaviour (left panel of Fig. 4), with no large deviations localized in specific traps. This might suggest that the local variability at trap level is well captured by the covariates and the temporal effect. On the contrary, there is a clear separation between the Central Macedonia region, with predominantly negative values, and the South-West part of Crete and Western Greece. This disparity could be influenced by proximity to the coast or differences in elevation. Moreover, differences in land use/land cover and (possibly) differences in *Culex* species may also have an impact. The spatial standard deviations (right panel of Fig. 4) do not show any significant spatial pattern. It is worth noting that the values are lower in the Central Macedonia region and in parts of Western Greece and Crete, where the majority of traps are located.

### Model performance and predictive ability

**Figure 5:**
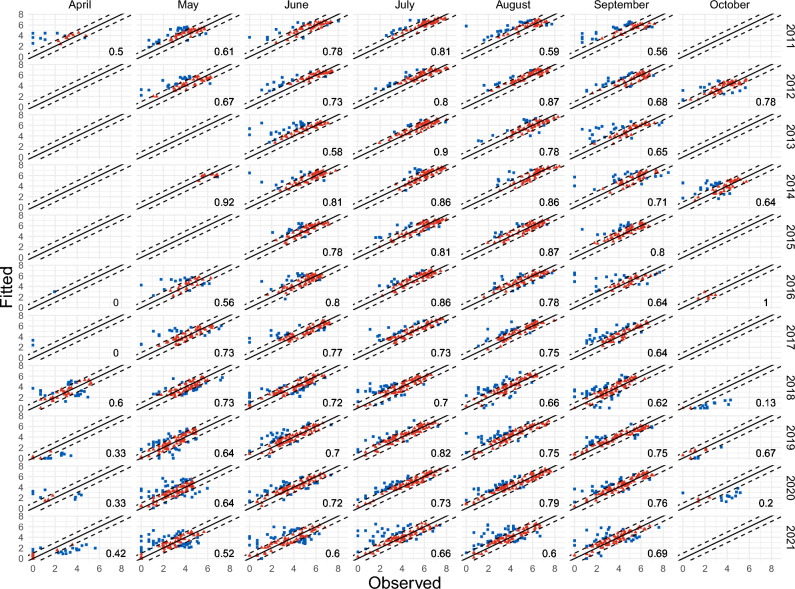
Observed vs fitted logarithmic mosquitoes abundance values, stratified by month and year. The dashed lines correspond to the $\pm 1$ interval, the red (triangle) dots represent the amount of pair that fall within these limits, while the number at the bottom-right corresponds to the proportion of observations that fall in the interval.

Our model provides a better fit in the months from June to September, where there is a higher availability of data (Fig. 5). Conversely, modeling the months of April and October proves to be more challenging due to the limited amount of data available, both in terms of calendar year and trap location. The difficulty in modeling the years 2018 and 2021 is also evident and reflects the high variability previously discussed in the temporal effects.

In order to check the robustness of the model in terms of its predictive ability, we evaluated MAE and mean-squared error (MSE, in log scale) and the mean-absolute error (MAE), defined as

$$\begin{aligned} {\text {MAE}} = \frac{1}{n} \sum _{1}^{n}|y_{i} - \hat{y}_{i}|, \end{aligned}$$

where $y_{i}, \hat{y}_{i}$ are the class indices (see^20^ for the details), both in train and test set in a leave one out procedure. In particular, in the first part of Table 1, we report the errors computed by using one year at a time as test set, and the remaining years as training. Similarly, in the second part of Table 1, we report the errors computed by using one region at a time as test set, and the remaining years as training. Overall, the results shows a convincing robustness of the model, with test errors always below 1.5 for MAE and 1.6 for MSE, except for the years 2018 and 2021 (exceptional years as previously described). As for the regions, the results are comparable but less clear. This may be due to two main reasons: first, the number of observation is disproportionally biased towards Central Macedonia, with an expected increase in the errors when this is used as test set; second, unlike for the years for which the priors are independent, in the spatial dimension is present a distance dependence correlation that might influence the results when the traps of a whole region are removed from the training set.

**Table 1:** Training and prediction errors (Mean Absolute Error and Mean Squared Error) for different years (for each calendar year, the model is estimated using all the years except the considered one, used for prediction) and different regions.

Year	MAE train	MAE	MSE train	MSE
2011	1.23	1.40	1.23	1.53
2012	1.24	1.13	1.27	0.93
2013	1.23	1.29	1.22	1.54
2014	1.23	1.23	1.22	1.28
2015	1.25	1.08	1.27	0.87
2016	1.22	1.37	1.22	1.54
2017	1.24	1.28	1.25	1.20
2018	1.21	1.82	1.22	2.08
2019	1.26	1.29	1.28	1.25
2020	1.24	1.22	1.26	1.18
2021	1.15	1.88	1.09	2.70

Region	MAE train	MAE	MSE train	MSE
Central Macedonia	1.13	1.62	1.39	1.87
Crete	1.32	0.59	1.30	1.07
Thessaly	1.25	1.48	1.27	1.55
Western Greece	1.21	1.59	1.20	1.93

**Figure 6:**
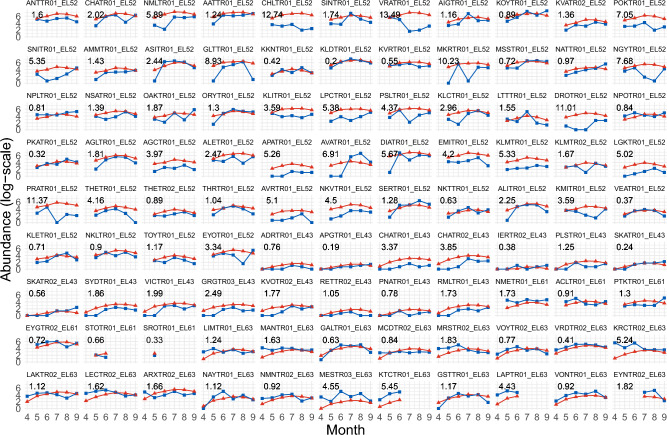
Observed (blue/squares) vs predicted (red/triangles) values for the year 2021 (model estimated using data from 2011-2020), stratified by trap, while the number at the top-left corresponds to the MSE. The traps are ordered as in Fig. 1.

In Fig. 6, we present the predicted time series for the year 2021. In terms of error, the year 2021 seems to be the most challenging to predict, possibly due to external factors not included in the data. Nonetheless, even though we obtain high MSEs for some traps, the models is able to capture the overall behaviour of the majority of the traps. As a comparison, we present the same plot of predicted time series for the year 2015, i.e. the year with the lowest MSE, in Supplementary Figure S2. In this case, the MSEs are considerably lower and the model performs well for the majority of the traps, with the predicted time series closely following the observations. The overall behaviour is quite consistent, with the model being able to predict a bell-shaped curve with peak mosquito abundance in July and August and lower values in the surrounding months, even when the time series have limited data. However, for the traps with higher error values, there are often peculiar patterns in the mosquito abundance, such as unexpected low numbers in certain months (see for example traps DROTR01 EL52 and VRATR01 EL52) or fluctuating abundance (see for example MKRTR01 EL52 and PRATR01 EL52). Such local pattern may depend on exogenous characteristics that would deserve a deeper understanding.

## Discussion

In this study, a hierarchical Bayesian spatio-temporal model was employed on WNV surveillance data from Greece to investigate the impact of climatic and environmental factors on the *Culex* mosquito population abundance. The potential of this approach was also to explore the possibility of using the gained knowledge to predict mosquitoes abundance.

Our results suggested that temperature is a major driver of the mosquito population, which is consistent with previous publications, exploring this association^19,21–27^. Paz & Albersheim^21^, observed a positive association between abundance of *Culex pipiens* and the average temperature with two-week lags. This is indirectly captured in our model by the number of grow days in the previous month, that has the second most important positive association. Similarly, in a study on WNV vectors’ abundance, Bisanzio et al.^22^ reported a significant positive relation between weekly abundance of *Culex pipiens* and *Culex modestus* with the temperature in the week before trapping. This lags might be explained following the impact that temperatures have on mosquito development: Soh & Aik^27^ observed a positive association between temperature and *Culex* larval habitats in Singapore. Similarly, Gardner et al.^24^, specifically on WNV vectors, and Ukubuiwe et al.^28^, on general abundance of *Culex quinquefasciatus*, reported a faster immature development, as well as faster larval growth and shorter duration of immature phase in warmer rearing water, respectively. Observing the abundance of WNV vectors *Culex pipiens*, Ruybal et al.^19^ reported a monotonic and non-linear inverse relation between adult female development and temperature, as well as a direct relation between larval survival and temperature under 27 $^\circ$C. In addition, the same authors observed a negative relation between adult female and larval survival, and higher temperature^19^. Similarly, Ciota et al.^17^, in a study on factors driving the general abundance of *Culex pipiens*, *Culex quinquefasciatus*, and *Culex restuans*, observed a positive association between mosquito development and increased temperatures, with the association weakening after 24$^\circ$C. These two latter results are especially important, as they allows uncertainty over the role of climate change in the distribution and epidemiology of mosquito-borne diseases. In fact, it is common concern that climate change could increase the distribution areas of arbovirus and their vectors, as well as the prevalence of arboviral diseases. However, the number of CWD and CDD, which serve as proxies for soil moisture, also play a synergistic role in shaping the mosquito population. High soil moisture levels are positively associated with mosquito survival due to the high surface area to volume ratio of mosquitoes, which makes them susceptible to desiccation^29^. Water availability is another important factor that positively affects mosquito abundance. Although rain might prove beneficial, providing natural and artificial water bodies for female mosquitoes to lay their egg, heavy rainfall can be detrimental to the mosquito population, as it can flush away breeding habitats and immature individuals, especially among *Culex pipiens*^24,30^, as well as reducing daily survival rates among adults^31^. Our study results support these hypotheses, showing a negative association between high levels of rainfall both in the current as well as in the month before trapping, and mosquito abundance. Similar results were found by Soh & Aik^27^, who observed a negative association between rainfall and *Culex* larval habitats in Singapore. However, there is not yet consensus on this association. Bisanzio et al.^22^ reported a positive relation between cumulative rain in the 10 days before trapping and weekly abundance of *Culex pipiens*. Reisen et al.^32^ found both a positive and negative correlation between rainfall and abundance of the population of *Culex tarsalis* in different regions of California. A possible explanation for the duplicity of this association was provided by Valdez et al.^33^, whom observed in their model that elevated heterogeneity in daily levels of rainfall reduces the dependence of *Culex quinquefasciatus* abundance on rainy days. Another possible explanation was reported by Carrieri et al.^34^, who observed that, in urban environments, breeding site are mostly related to human environments and, therefore, rainfall does not influence the seasonal trends of mosquito abundance. Furthermore, following Wang et al.^23^, the impact of rainfall is clearly lower than the one of temperature, and—in addition—develops over longer time (these authors suggest 35 days before capture).

Other climatic and environmental conditions have shown to be associated with mosquito abundance in our model: NDWI and NDVI presented a positive association with mosquito population, whereas CWD and CDD in the previous month, and the average wind speed presented a negative association. A huge number of dry days, together with an high amount of rainfall in the previous month does not favour large *Culex* abundances. This results suggest that non-extreme drought/rainfall conditions are more likely to enhance *Culex* abundance (due to, e.g., development of small-to-medium-sized artificial water bodies). Just as per temperature and rainfall, similar results have been already presented, e.g., NDVI have been already found to be positively associated with the abundance of *Culex modestus*^22^, whereas wind speed was associated if not directly with mosquito abundance, with their WNV infection rate^35^.

**Figure 7:**
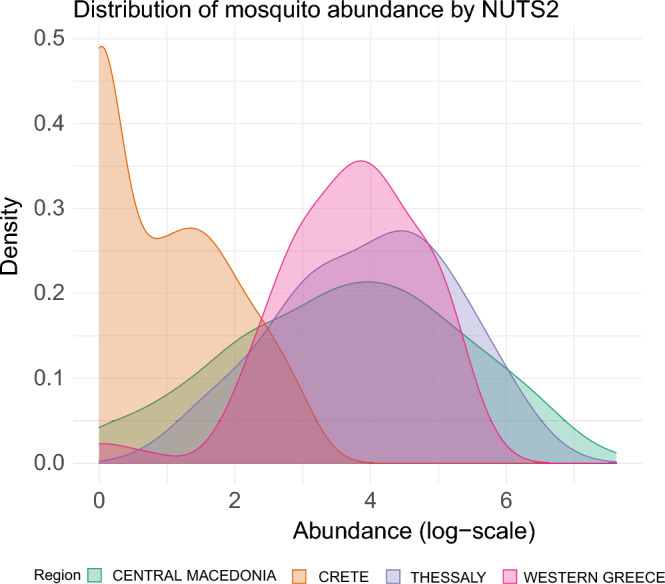
Distribution of observed mosquitoes (log-scale), stratified by region.

Concerning its ability to predict the abundance of mosquitoes, our model successfully captures the bell-shaped curve of mosquito abundance while maintaining enough flexibility to adapt to the different spatial characteristics. For a large portion of the traps, the model predictions align with the observed abundance. However, traps that display more oscillatory behavior remain challenging to model and it is uncertain whether these fluctuations are due to exceptional weather conditions or unrepresentative trap abundances. For this reason, perhaps a more homogeneous distribution of trap sites would provide help to better uncover the effect of the gradient of temperature, soil moisture, and rainfall on WNV-transmitting *Culex* mosquitoes abundance. In addition, it is worth noting the quite unique behavior in Crete (Fig. 7), which is characterized by a large number of low values, especially during April and May: unlike other regions that have a similar distribution shape, Crete exhibits a significantly left-skewed distribution. These spatial variability of Crete could be attributed to factors such as elevation or proximity to the sea; and though the spatial component of the model captures this behavior to a certain extent, capturing the very small values of abundance in just one region might require a more complex approach.

Our study has certain limitations. Firstly, the data presents several challenges, including spatial heterogeneity, with two-thirds of the traps located in the region of Central Macedonia, and a significant amount of disproportionate information availability for a huge portion of traps and years. This potential source of selection bias for the observed areas results from the origin of the data that we used. The areas, which were more explored (e.g., more traps for more years), were the ones with higher prevalence of WNV disease. It is important to keep this in mind when interpreting our results: though the model might apply to general *Culex pipiens* population abundance, we cannot exclude an influence of the distribution of the trapping sites on our observations, and therefore we suggest to always contextualizing the reported estimate to WNV surveillances. On our side, to address these challenges, we define a hierarchical model that is able to capture the spatio-temporal autocorrelation characteristics of the data, possibly due to unmeasured variables (e.g. different availability of hosts due to nesting or emigration of preferred host species, development of stronger defensive behaviors by potential hosts, shift in feeding patterns^36^). The model also allows appropriately dealing with the huge amount of missing values in the observed mosquito abundances, by borrowing information across both the temporal and the spatial dimension (there is no direct imputation as the model is able to handle missing data by combining the information from the nearby similar traps). In addition, it is paramount to understand the generalizability of the data: as the model is based on the mosquito abundance in traps, inevitably the amount of mosquitoes that are predicted in other areas represent the abundance that would be found in a trap, if one was set at the specific location, rather than the overall mosquito abundance. A limitation to this approach might be that our results were not adjusted by trap type. Following^37^, different mosquito traps perform better in different context, also related to climatic and environmental factors. Hence, the type of traps used for the collection of mosquito might influence some of the results we observed.

We considered that a more thorough understanding of real-world setting mosquito population dynamic together with an improved ability to predict mosquitoes’ population abundance would be relevant both to support policy makers in their evaluation of optimal planning of preventive interventions, as well as in enhancing preparedness and anticipation of the potentially changing risk of virus transmission resulting from climate variability. We observed that the analysis of the impact of climatic and environmental factors on mosquito population abundance could be challenging due to the uneven distribution of trap locations and the high variability in captured mosquitoes. In fact, despite representing a fairly complete and extensive cross-section of the mosquito population in Greece over a decade, the considered data remains very complex to study due to the high amount of missing values. A better understanding of these key factors is crucial for improving statistical models for population dynamics and could help in the development of early warning systems to reduce human exposure through vector control.

Our results provide strong evidence that climatic factors play a significant role in shaping the dynamics of the mosquito population; however, it remains challenging to fully understand the underlying processes. This complexity is compounded perhaps by the intrinsic bipartite life cycle of mosquitoes, where larvae develop in aquatic habitats and adults are terrestrial^38^, and which could benefit from further research. In addition, our results could support future studies aiming at disentangling the potential relationship between mosquito abundance and incidence of WNV disease. Nevertheless, on a general level, the results evaluated using the mean absolute error are similar to previous results on modelling mosquito abundance^20^. The approach proposed in this study shows promising results and provides insights into the role of climatic and environmental factors on mosquito population dynamics.

## Methods

### Data collection

The purpose of this analysis is to examine the seasonal and spatial distribution of the mosquito population in Greece and its association with various environmental and climatic conditions. The data consist of weekly observations from 2011 to 2021 obtained at 565 unique trap locations. Data on mosquito abundance were obtained from EcoDevelopment S.A. a private company performing mosquito surveillance and implementing mosquito control projects in Greece. Data on environmental factors are obtained from the EarlY WArning System for Mosquito borne disease (EYWA) dataset, developed as part of the EuroGEO Action Group ”Earth Observation for Epidemics of Vector-borne Diseases”, under the coordination of the National Observatory of Athens/BEYOND Centre of Earth Observation Research and Satellite Remote Sensing. The EcoDevelopment dataset, which is part of the EYWA dataset, contains *Culex* specific information based on weekly observation of 126 mosquito-traps, which were installed in 565 unique trap locations over Greece. The information include *Culex* abundance, defined as the total number of *Culex* mosquitoes caught in the trap placement; *number of pools*, defined as the number of mosquito pools that were sent to be analyzed for the presence of WNV; *pool size*, defined as the number of mosquitoes per pool; the *type of trap* used; *WNV positivity*, defined as the mosquito infection rate in each pool and the *number of mosquito breeding sites* within a buffer of 1km around sampling/trapping sites. The EYWA dataset contains also geographical information, in particular the definition of the collection site at Nomenclature of Territorial Units for Statistics (NUTS)-3 and Local Administrative Units (LAU)-2 level. In addition, a list of environmental features are available either at pixel level or at a specific resolution:

- the normalized difference vegetation, water, moisture, and built-up indices (NDVI, NDWI, NDMI, NDBI, respectively), both in the specific pixel in which the trap was set, as well as the average value (and standard deviation) of the neighboring pixels (3x3),
- the mean land surface temperature for the day and night, as well as for the months of January, February, March, April obtained from the Moderate-resolution Imaging Spectroradiometer (MODIS);
- the magnitude of wind (max, min, and mean),
- the accumulated precipitation counting towards one and two weeks before the date of placement of the traps, as well as counting from the first of January of each year.
- the number of waste water treatment facilities within a buffer of 1000 m around sampling/trapping sites,
- the distance of combination of breeding site length and length of watercourses of national hydrological data within a buffer zone of 1000 m around each sampling/trapping site,
- the total area of temporarily inundated areas (polygons) within a buffer zone of 1000 m from each sampling/trapping site or human case,
- the total area of wetlands (polygons) within a buffer zone of 1000 m from each sampling/trapping site or human case,
- the mean Distance of sampling/trapping site within a buffer of 1000 m from coastline,
- the mean elevation (resolution = 12.5 m), within a buffer of 1000 m around trapping sites,
- the mean slope (12.5 m), within a buffer of 1000 m around trapping sites,
- the mean aspect (12.5 m), within a buffer of 1000 m around trapping sites,
- the mean flow accumulation within a buffer of 1000 m around trapping sites,
- the distance of sampling/trapping sites within a buffer zone of 500 m from nearest surface water polygon.

Meteorological data come from the European Centre for Medium-range Weather Forecasts (ECMWF)’s ERA5 the fifth generation atmospheric reanalysis of the global climate. Before being included in the model, temperature data were smoothed to eliminate fluctuations with a Kolmogorov-Zurbenko filter^39^. The metereological data include temperature average (*TAVG*) and rainfall (*RAIN*) observed daily at municipality level. In order to capture potential more complex behaviors, we derived a list of additional features. The number of wet days (rainfall $> 0.5$ mm per day); the number of dry days (rainfall $\le 0.5$ mm per day); the number of grow days (daily temperature average $>14.3$ C); the maximum number of consecutive wet days ; maximum number of consecutive dry days; the maximum number of consecutive grow days; the cumulative number of grow days since January. Moreover, we also considered the time-lagged version of the previous variables: average temperature in the previous month; the average rainfall in the previous month; the number of wet days in the previous month; the number of dry days in the previous month; the number of grow days in the previous month; the maximum number of consecutive wet days; the maximum number of consecutive dry days; the maximum number of consecutive grow days.

### Data preparation

The completeness in the data is quite heterogeneous, with a variety of traps having missing information for most years both in the outcome variable (mosquito abundance), as it is clear from Fig. 1, and in the covariates. In order to improve the consistency of the data, the original EcoDevelopment S.A. dataset was first filtered to include only records with non-missing values for mosquitoes abundance. Moreover, the predictors with more than 5% of missing values were removed (land surface temperature, watercourses, maximum and minimum wind, aspect, slope, flow). We included only the observations in the months from April to October, as well as traps with less than four years of observations. This step does not drastically change the nature of the data, since the aim of the analysis is to capture the long term behavior of the mosquito population in the period of maximum abundance (from late spring to early autumn).

Finally, given the high number of variables and the possible issue of strong correlation among them, we decided to screen the dataset in order to remove pairs of variables with Spearman correlation higher than 0.75. The final set of covariates include 12 covariates: *NDVI* (proxy for vegetation density) and *NDWI* (proxy for water content) averaged over the neighboring pixels ($3\times 3$); *WIND* (average wind speed), *TAVG* (average temperature in C) and *RAIN* (average amount of precipitations in mm) averaged by month; the three additional features *CWD* (maximum consecutive number of wet days), *CDD* (maximum consecutive number of dry days) and *CGD* (maximum consecutive number of grow days); the lagged versions of the previous variables *CWD_l1*, *CDD_l1*, *CGD_l1* and *RAIN_l1*. The final set of covariates, together with the inclusion of the spatio-temporal component described in details the next section, cover all the aspects of climate and environmental factors under study. The obtained dataset comprises of 4077 observations from 126 unique traps and covers the period from 2011 to 2021.

### Model

We model the mosquito abundance *y*(*s*, *j*, *t*) observed at site *s*, year *j* and month *t*. We assume to have a set of traps $\{s_i \}_{(i=1,\ldots ,I)}$. For each *i*, the set of indices $k \in A_{i}$, with $A_{i}$ comprising the years for which we have observations for the trap *i*. Moreover, the set of indices $j \in M_{i_{k}}$, with $M_{i_{k}}$ comprising the months of year *k* for which we have observations for the trap *i*. The model assumes the mosquito abundance to be a realization of a Poisson process, (with expected number of cases $E_i$ taken to be the average mosquito abundance by trap), with a mean comprising a spatio-temporal component and a set of covariates.

The model is estimated using Integrated Nested Laplace approximations^40,41^, using the R-software package INLA (version 22.04.16, www.r-inla.org) with default priors for the coefficients. This Bayesian approach provides an intuitive and explicit estimation of model uncertainty, accounting for spatially and temporally correlated errors. Similar Bayesian spatio-temporal processes have been already used in the epidemiological literature to model, for instance, HIV mortality, thanks to their flexibility^42,43^. The hierarchical spatio-temporal process is the following:

$$\begin{aligned} y(s,j,k)&\sim {\text {Poisson}}(\lambda ) \\ \lambda&= f(s)+ \gamma (j)+ \xi (k) + X_{sjk}^{\top } \beta \end{aligned}$$

where $\beta$ is the vector of coefficients for the fixed effects. The spatial effect *f* is modelled via a Gaussian random field with a Matérn covariance function, using an approximated stochastic partial differential equations approach^40,41^. The covariance between measurements taken at two trap sites $(s_{i}, s_{i'})_{i \ne i'}$, separated by a distance $d = ||s_{i} - s_{i'}||$, is given by

$$\begin{aligned} {\displaystyle C_{\nu }(d)=\sigma ^{2}{\frac{2^{1-\nu }}{\Gamma (\nu )}}{\Bigg (}{\sqrt{2\nu }}{\frac{d}{\rho }}{\Bigg )}^{\nu }K_{\nu }{\Bigg (}{\sqrt{2\nu }}{\frac{d}{\rho }}{\Bigg )},} \end{aligned}$$

where $\Gamma$ is the gamma function, $K_{\nu }$ is the modified Bessel function of the second kind, and $\rho$ and $\nu$ are positive parameters of the covariance. The estimated posterior values for $(\theta _1, \theta _2)$ provided in the Results Section correspond to the transformations

$$\begin{aligned} \theta _1 = \log (1/\sigma ^2) , \quad \theta _2 = \log (\rho ) . \end{aligned}$$

The range of the spatial process is controlled by parameter $\rho$. Higher values of the parameter induce a faster decay in the correlation with distance, which imply a small range spatial process. On the contrary, lower values will indicate a spatial process with a large range. The parameter $\nu$ controls smoothness of the spatial process. Finally, the parameter $\sigma ^2$ a general scale parameter. The first temporal component $\gamma$ (for months) consists of a first order difference prior, so that given a grid $\{ \kappa _0, \ldots , \kappa _K \}$, we have

$$\begin{aligned} \gamma (\kappa _{i+1}) - \gamma (\kappa _{i}) \sim \mathcal {N}(0, \tau _{\gamma }), \, i = 1, \ldots , K-1. \end{aligned}$$

Here the we use a regular grid that corresponds to the months considered. This prior is the Bayesian equivalent of spline smoothing, so that large differences on the effect of two consecutive months are penalized. The second temporal component $\xi$ (for years) consists of a Gaussian prior with independent components, since there is no clear evidence on the dependence among years. Moreover, we use non-informative priors for the coefficients of the linear part. All posterior expectations and probabilities were estimated using 1000 samples.

The model and the predictions are obtained using a high-resolution Delaunay triangulation mesh of the study area (see Supplementary Figure S3), constructed starting from the official boundaries obtained from Eurostat (https://ec.europa.eu/eurostat/web/gisco/geodata). The mesh is obtained via the function inla.mesh.2d of the package INLA, using parameters max.edge$= (1, 5)\cdot 0.95$, cutoff$= 0.06$, min.angle$= 30$.

### Supplementary Information

Supplementary Information.

## Data Availability

The data on mosquito abundance in traps are available from EcoDevelopmet S.A. but restrictions apply to the availability of these data, which were used under license for the current study, and so are not publicly available. Data are however available from the authors upon reasonable request and with permission of EcoDevelopmet S.A. The other data used and/or analysed during the current study are available from the corresponding author on reasonable request.
