# Supplementary material for: Effects of climatic and environmental factors on mosquito population inferred from West Nile virus surveillance in Greece
Source: Sci Rep. 2023 Nov 1;13:18803. doi: 10.1038/s41598-023-45666-3 (PMC10620416; doi:10.1038/s41598-023-45666-3)
Supplement: Supplementary file 1 — Supplementary Information. [file 41598_2023_45666_MOESM1_ESM.pdf]

Supplementary Material - Effects of climatic and environmental factors on mosquito population inferred from West Nile virus surveillance in Greece

Federico Ferraccioli[1,2], Nicola Riccetti[1], Augusto Fasano[1,3], Spiros Mourelatos[4], Ioannis Kioutsioukis[5,*], and Nikolaos I. Stilianakis[1,6]

[1] *European Commission, Joint Research Centre (JRC), Via E. Fermi 2749, 21027 Ispra, VA, Italy*
[2] *Current: Department of Statistical Sciences, University of Padova. Via C. Battisti 241, 35121 Padova, PD, Italy*
[3] *Current: Department of Statistics, Catholic University of the Sacred Heart, Largo A. Gemelli, 20123 Milano, MI, Italy*
[4] *EcoDevelopment SA, Filiro, 57010 Thessaloniki, Greece*
[5] *Department of Physics, University of Patras, 26504 Rio, Greece*
[6] *Department of Biometry and Epidemiology, University of Erlangen-Nuremberg, Waldstraße 6, 91054 Erlangen, Germany*
[*] *Corresponding author: Ioannis Kioutsioukis, kioutio@upatras.gr*

S1 Results and implementation details

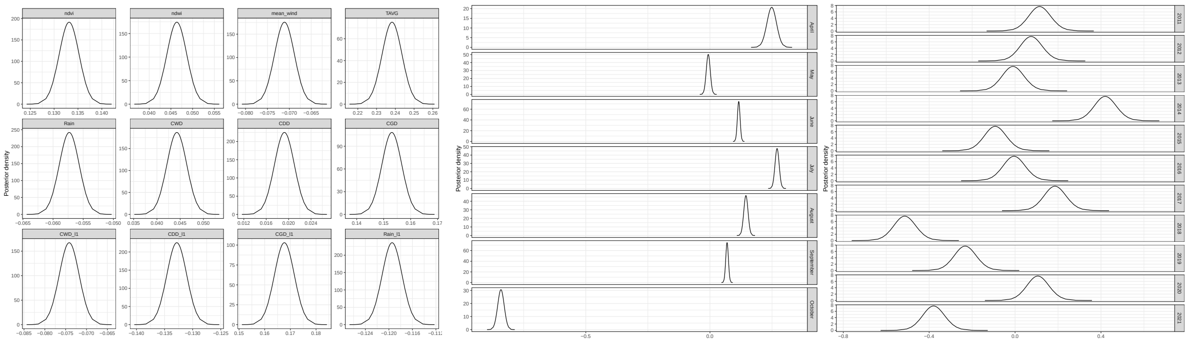

Supplementary Figure S1: Left: Posterior distributions for the fixed effect. Center: Posterior distributions for the month random effect. Right: Posterior distributions for the year random effect.

The mesh used for the estimation (see Supplementary Figure S3) was constructed with the function `inla.mesh.2d` starting from the country boundaries (extracted from Eurostat), using parameters `max.edge`$= (1, 5) \cdot 0.95$, `cutoff`$= 0.06$, `min.angle`$= 30$.

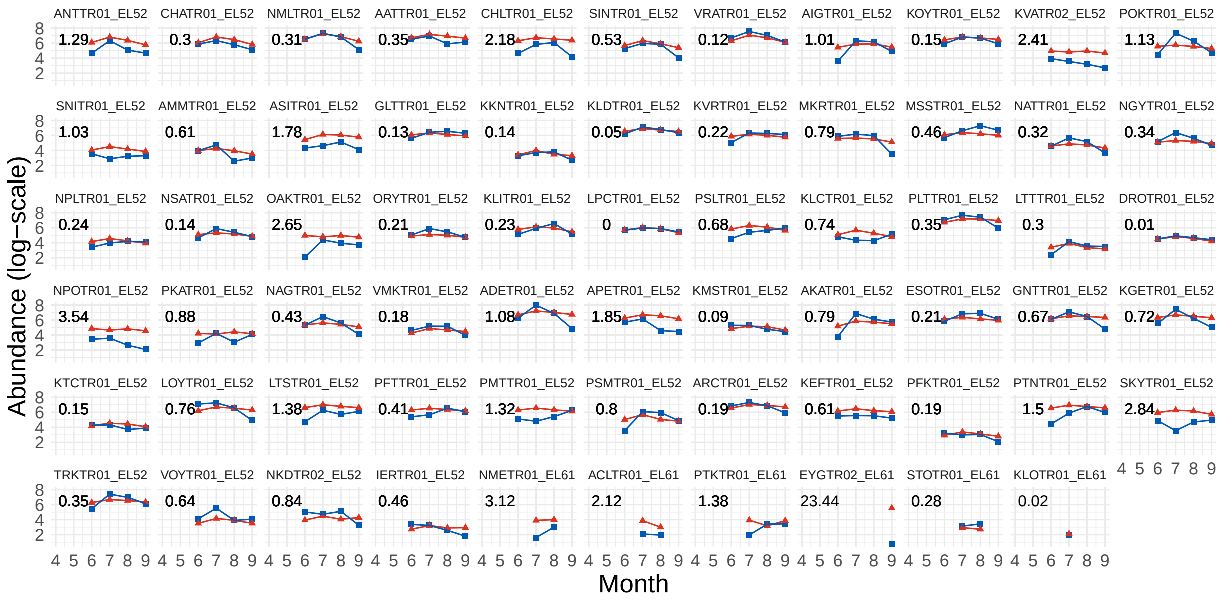

Supplementary Figure S2: Observed (blue/squares) vs predicted (red/triangles) values for the year 2015 (model estimated using data from the other years), stratified by trap, while the number at the top-left corresponds to the MSE. The traps are ordered as in Figure 1.

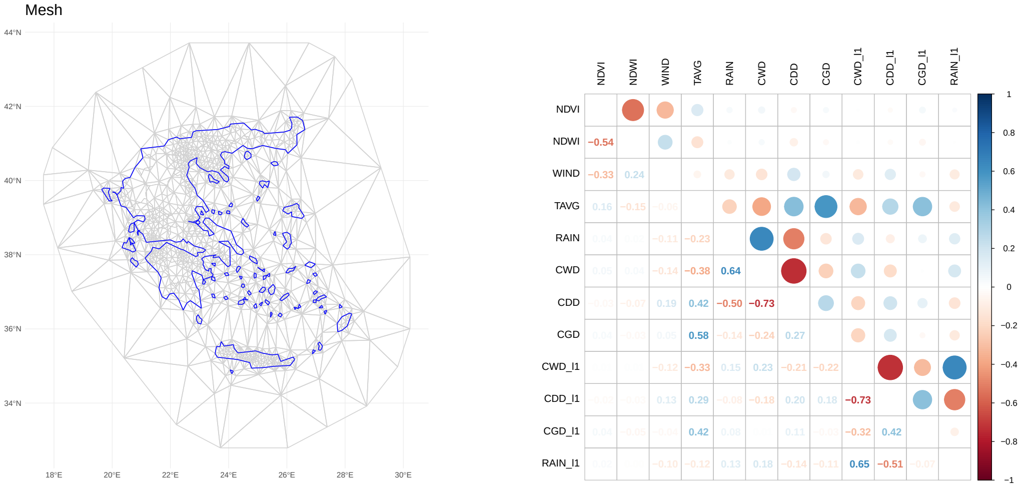

Supplementary Figure S3: Left: mesh used in the estimation. Right: correlation of the input variables used in the model.

S2 Descriptive tables

Supplementary Table S1: Summary statistics for all the traps.

trap_id	nuts2_name	Abundance (avg)	SD	N. of obs.	N. of years
AATTR01_EL52	CENTRAL MACEDONIA	752.55	831.43	56	11
ADETR01_EL52	CENTRAL MACEDONIA	879.37	824.86	30	6
AGCTR01_EL52	CENTRAL MACEDONIA	47.12	45.39	26	5
AGLTR01_EL52	CENTRAL MACEDONIA	225.56	199.35	45	9
AIGTR01_EL52	CENTRAL MACEDONIA	227.38	215.35	55	11
AKATR01_EL52	CENTRAL MACEDONIA	255.76	262.53	29	6
ALETR01_EL52	CENTRAL MACEDONIA	477.23	562.92	26	5
ALITR01_EL52	CENTRAL MACEDONIA	130.74	120.53	23	5
AMMTR01_EL52	CENTRAL MACEDONIA	43.00	55.21	54	11
ANTTR01_EL52	CENTRAL MACEDONIA	437.95	403.52	57	11
APATR01_EL52	CENTRAL MACEDONIA	14.27	20.14	26	5
APETR01_EL52	CENTRAL MACEDONIA	485.17	486.98	30	6
ARCTR01_EL52	CENTRAL MACEDONIA	745.79	675.69	28	6
ASITR01_EL52	CENTRAL MACEDONIA	250.48	172.06	54	11
AVATR01_EL52	CENTRAL MACEDONIA	71.58	158.50	26	5
AVRTR01_EL52	CENTRAL MACEDONIA	16.20	16.01	25	5
CHATR01_EL52	CENTRAL MACEDONIA	479.44	418.79	57	11
CHLTR01_EL52	CENTRAL MACEDONIA	488.46	557.19	56	11
DIATR01_EL52	CENTRAL MACEDONIA	547.08	441.51	26	5
DROTR01_EL52	CENTRAL MACEDONIA	72.04	116.95	47	10
EMITR01_EL52	CENTRAL MACEDONIA	325.81	336.40	26	5
ESOTR01_EL52	CENTRAL MACEDONIA	411.41	323.24	29	6
EYOTR01_EL52	CENTRAL MACEDONIA	111.47	65.81	19	4
GLTTR01_EL52	CENTRAL MACEDONIA	363.72	285.40	54	11
GNTTR01_EL52	CENTRAL MACEDONIA	539.52	356.74	29	6
IERTR01_EL52	CENTRAL MACEDONIA	17.19	17.64	21	5
KEFTR01_EL52	CENTRAL MACEDONIA	448.46	502.98	28	6
KGETR01_EL52	CENTRAL MACEDONIA	555.83	431.58	29	6
KKNTR01_EL52	CENTRAL MACEDONIA	30.48	32.59	54	11
KLCTR01_EL52	CENTRAL MACEDONIA	163.84	156.21	50	10
KLDTR01_EL52	CENTRAL MACEDONIA	622.20	563.35	54	11
KLETR01_EL52	CENTRAL MACEDONIA	25.86	22.52	21	4
KLITR01_EL52	CENTRAL MACEDONIA	283.94	243.81	53	11
KLMTR01_EL52	CENTRAL MACEDONIA	85.31	64.93	26	5
KLMTR02_EL52	CENTRAL MACEDONIA	20.12	11.80	26	5
KMITR01_EL52	CENTRAL MACEDONIA	49.91	60.15	23	5
KMSTR01_EL52	CENTRAL MACEDONIA	118.83	170.01	30	6

trap_id	nuts2_name	Abundance (avg)	SD	N. of obs.	N. of years
KOYTR01_EL52	CENTRAL MACEDONIA	608.91	513.98	55	11
KTCTR01_EL52	CENTRAL MACEDONIA	64.90	55.79	29	6
KVATR02_EL52	CENTRAL MACEDONIA	88.85	116.57	55	11
KVRTR01_EL52	CENTRAL MACEDONIA	337.06	244.32	54	11
LGKTR01_EL52	CENTRAL MACEDONIA	18.38	25.62	26	5
LOYTR01_EL52	CENTRAL MACEDONIA	592.66	413.20	29	6
LPCTR01_EL52	CENTRAL MACEDONIA	251.25	249.57	53	11
LTSTR01_EL52	CENTRAL MACEDONIA	646.31	906.06	29	6
LTTTR01_EL52	CENTRAL MACEDONIA	27.80	33.25	49	10
MKRTR01_EL52	CENTRAL MACEDONIA	207.07	193.91	54	11
MSSTR01_EL52	CENTRAL MACEDONIA	407.43	329.16	54	11
NAGTR01_EL52	CENTRAL MACEDONIA	196.87	156.95	31	7
NATTR01_EL52	CENTRAL MACEDONIA	87.33	82.06	54	11
NGYTR01_EL52	CENTRAL MACEDONIA	142.63	143.71	54	11
NKDTR02_EL52	CENTRAL MACEDONIA	75.05	73.44	22	5
NKLTR01_EL52	CENTRAL MACEDONIA	89.00	87.24	21	4
NKTTR01_EL52	CENTRAL MACEDONIA	26.42	19.49	24	5
NKVTR01_EL52	CENTRAL MACEDONIA	79.52	62.22	25	5
NMLTR01_EL52	CENTRAL MACEDONIA	720.19	748.25	57	11
NPLTR01_EL52	CENTRAL MACEDONIA	56.39	56.41	54	11
NPOTR01_EL52	CENTRAL MACEDONIA	79.79	72.27	47	10
NSATR01_EL52	CENTRAL MACEDONIA	126.65	112.34	54	11
OAKTR01_EL52	CENTRAL MACEDONIA	92.54	97.08	54	11
ORYTR01_EL52	CENTRAL MACEDONIA	119.48	103.65	54	11
PFKTR01_EL52	CENTRAL MACEDONIA	19.54	22.63	28	6
PFTTR01_EL52	CENTRAL MACEDONIA	487.14	454.00	29	6
PKATR01_EL52	CENTRAL MACEDONIA	51.81	40.56	47	10
PLTTR01_EL52	CENTRAL MACEDONIA	978.56	1037.72	50	10
PMTTR01_EL52	CENTRAL MACEDONIA	457.83	370.44	29	6
POKTR01_EL52	CENTRAL MACEDONIA	227.67	266.67	55	11
PRATR01_EL52	CENTRAL MACEDONIA	124.85	161.27	26	5
PSLTR01_EL52	CENTRAL MACEDONIA	327.04	312.03	53	11
PSMTR01_EL52	CENTRAL MACEDONIA	174.72	158.79	29	6
PTNTR01_EL52	CENTRAL MACEDONIA	665.39	496.30	28	6
SERTR01_EL52	CENTRAL MACEDONIA	120.44	132.66	25	5
SINTR01_EL52	CENTRAL MACEDONIA	274.14	253.57	56	11
SKYTR01_EL52	CENTRAL MACEDONIA	326.18	275.75	28	6
SNITR01_EL52	CENTRAL MACEDONIA	48.00	64.14	55	11
THETR01_EL52	CENTRAL MACEDONIA	46.96	32.26	26	5
THETR02_EL52	CENTRAL MACEDONIA	18.04	15.89	26	5
THRTR01_EL52	CENTRAL MACEDONIA	62.42	55.50	26	5
TOYTR01_EL52	CENTRAL MACEDONIA	20.95	19.40	20	4
TRKTR01_EL52	CENTRAL MACEDONIA	569.54	431.96	28	6
VEATR01_EL52	CENTRAL MACEDONIA	21.74	12.82	23	5
VMKTR01_EL52	CENTRAL MACEDONIA	90.13	66.53	31	7
VOYTR01_EL52	CENTRAL MACEDONIA	49.04	53.15	28	6
VRATR01_EL52	CENTRAL MACEDONIA	655.96	682.06	56	11

trap_id	nuts2_name	Abundance (avg)	SD	N. of obs.	N. of years
ADRTR01_EL43	CRETE	2.26	2.38	23	4
APGTR01_EL43	CRETE	1.91	4.55	23	4
CHATR01_EL43	CRETE	8.70	14.07	23	4
CHATR02_EL43	CRETE	19.96	22.08	23	4
GRGTR03_EL43	CRETE	11.32	13.75	22	4
IERTR02_EL43	CRETE	1.04	1.36	23	4
KVOTR02_EL43	CRETE	9.14	11.48	22	4
PLSTR01_EL43	CRETE	4.57	4.69	23	4
PNATR01_EL43	CRETE	2.71	3.98	21	4
RETTR02_EL43	CRETE	2.36	2.80	22	4
RMLTR01_EL43	CRETE	8.76	11.41	21	4
SKATR01_EL43	CRETE	3.26	4.53	23	4
SKATR02_EL43	CRETE	1.87	2.46	23	4
SYDTR01_EL43	CRETE	11.48	11.65	23	4
VICTR01_EL43	CRETE	15.70	12.64	23	4
ACLTR01_EL61	THESSALY	36.65	47.42	23	6
EYGTR02_EL61	THESSALY	193.63	162.85	19	5
KLOTR01_EL61	THESSALY	5.75	7.87	8	4
NMETR01_EL61	THESSALY	35.29	34.07	24	6
PTKTR01_EL61	THESSALY	41.14	46.12	22	6
SROTR01_EL61	THESSALY	20.50	25.43	16	5
STOTR01_EL61	THESSALY	11.84	10.44	19	6
ARETR02_EL63	WESTERN GREECE	27.44	18.97	16	4
ARXTR02_EL63	WESTERN GREECE	89.90	121.35	21	4
EYNTR02_EL63	WESTERN GREECE	32.00	56.11	14	4
GALTR01_EL63	WESTERN GREECE	55.50	74.39	24	5
GSTTR01_EL63	WESTERN GREECE	34.24	26.67	17	4
KRCTR02_EL63	WESTERN GREECE	44.18	75.20	22	4
KTCTR01_EL63	WESTERN GREECE	20.37	32.01	19	4
LAKTR02_EL63	WESTERN GREECE	51.73	49.95	22	4
LAPTR01_EL63	WESTERN GREECE	46.88	47.60	17	4
LECTR02_EL63	WESTERN GREECE	68.59	57.96	22	4
LIMTR01_EL63	WESTERN GREECE	20.10	13.48	30	6
MANTR01_EL63	WESTERN GREECE	24.08	16.00	25	5
MCDTR02_EL63	WESTERN GREECE	18.83	21.15	23	4
MESTR03_EL63	WESTERN GREECE	18.45	35.06	20	4
MRSTR02_EL63	WESTERN GREECE	32.91	39.46	23	4
NAYTR01_EL63	WESTERN GREECE	24.14	32.74	21	4
NMNTR02_EL63	WESTERN GREECE	23.71	22.33	21	4
VONTR01_EL63	WESTERN GREECE	42.24	42.04	17	4
VOYTR02_EL63	WESTERN GREECE	26.61	28.23	23	4
VRDTR02_EL63	WESTERN GREECE	75.87	55.39	23	4
